# Oral health delivery in refugee camps in East Region of Cameroon

**DOI:** 10.4314/ahs.v23i2.70

**Published:** 2023-06

**Authors:** Agbor Michael, Kaptue Bruno, Acha-teku Tetu, Ernest Tambo, Keboa Mark, Naidoo Sudeshni

**Affiliations:** 1 Universite des Montagnes Faculte des Sciences de la Sante, Universite des Montagnes; 2 Faculty of health and biomedical sciences, Université des Montagnes- Cameroon; 3 McGill University Faculty of Medicine, Global Oral health; 4 Department of Community Dentistry, University of the Western Cape, Community Oral health South Africa

**Keywords:** Oral health care, periodontal diseases, refugees, refugee camps, Cameroon

## Abstract

**Background:**

Oral health care affects the quality of life and plays an essential role in the general health of vulnerable populations especially refugees. The purpose of the study was to evaluate the quality of oral health care delivery in the Gado-Badzeré refugee camp in the Eastern region of Cameroon.

**Methods:**

We carried out a cross-sectional study between January and July 2020 using a structured questionnaire in French and translated orally to Fulfulde language.

**Results:**

A total of 716 refugees from the Central African Republic with ages ranging from 6 to 81 years (29.3years ± 14.6 s.d), made up of 61.2% females, 378(52.8%) unemployed, 342(47.8%) married, 701(97.9%) Muslims, and 511(71.4 %) had no formal education participated. Oral health knowledge was significantly poor, 305 (42.6%) that consulted the health post for their oral health needs were not satisfied, 640(89.50%) had experienced toothache, 592(83.0%) needed restorative treatment, 709(99.0%) periodontal treatment and 215(30.0%) urgent needs like tooth extraction. There were no oral health facilities, no oral health personnel, no oral health outreach had ever been carried out in the camp, and oral pathologies were managed by nurses with medications.

**Conclusion:**

The quality of oral health care delivery in this camp was very poor. There is an absence of oral health workforce and basic primary oral health care facilities. Oral health knowledge was very poor and the treatment needs the refugees was very high.

## Introduction

Several studies from both developed and developing countries have reported a high frequency of oral diseases amongst refugee communities 1. Refugees are faced with issues of cultural, ethnic, linguistic and religious origins making the difficult to adapt to their new unplanned settlements. Under such situations, the contribution of the refugees is extremely crucial for the effective planning and implementation of appropriate oral health care programs 1.

Oral pathologies, notably dental caries is amongst the most prevalent health care problems worldwide 2, 3, 4. Recent reports indicate that refugees are highly exposed to poor oral health and have higher prevalence of oral pathologies compared to host populations 5, 6. In 2019, the population of refugees and asylum seekers was estimated at 30.2 million, with the majority hosted in neighbouring source countries 7.

Many factors can influence the health of refugees; notably their geographic origins, the living conditions in the refugee camp and accessibility to proper health care 8, 9. Poor oral health of refugees is attributable to various factors such as limited access to therapeutic and preventive dental care, lack of dental services in camps, low oral health literacy, cultural and psychological factors, limited financial resources, poor diet and limited access to portable water 10,11, 12. Poor oral hygiene, poor hygiene practices and the interactions of the above factors can aggravate the oral health status of refugees.

Poor oral health has negative impacts on the quality of life and increase the risk of chronic diseases 13. For example, the pain from toothache can disturb mastication and negatively impact proper nutrition, while bacteria from periodontal disease are associated with diabetes and cardiovascular diseases 13. The consequences of oral disease are even more important for refugees, a vulnerable population already facing daily challenges. It is often an urgent need in improving on the oral health of refugees who have presented with acute dental emergencies, especially patients in pain from toothache that had been suffering from for weeks 13, 14. Most refugees' camps do not have facilities for dental treatment because they are often considered as temporary settlements. But basic dental interventions like excavating and temporizing large carious lesions have been shown to relieve pain 14.

Africa hosts more than a third of refugees in the world but little have been reported about the health status of these refugees and there is paucity of literature on the oral health status of refugees in the continent 15. This is because efforts have been put in place for medical intervention for refugee populations; but issues related to oral health are yet to be seen as a priority 15. Additionally other diseases such as HIV/AIDS and malaria which are endemic in sub-Saharan Africa are listed as priority in the list of policy makers because of their morbidity and mortality which make them to receive a lot of funding and attention from the government.

In Cameroon, refugees come from neighbouring countries facing socio-political unrests notably, Nigeria and Central Africa Republic (CAR). Cameroon is the host of the largest number of displaced people from CAR, estimated at about 248,000. These refugees are accommodated in five refugee camps in the East Region of Cameroon. The Gado Badzeré refugee camp is the biggest among these camps and hosts approximately 25,701 refugees 7. Little have been reported on the oral health needs and care in growing number of refugee camps in Cameroon. Therefore, the objectives of our study were: (i) to assess the oral health knowledge and practices of refugees in Gado-Badzeré refugee camp, Cameroon; (ii) to assess the oral health status and treatment needs of refugees in Gado-Badzeré refugee camp, Cameroon; and (iii) to evaluate the oral health care facilities and workforce present in the camp.

## Methodology

A cross sectional descriptive study was carried out at the Gado-Badzeré refugee camp in the Lom and Djerem division in the Eastern Region of Cameroon from January 2020 to June 2020. This camp was selected because it is the largest camp that has hosted refugees for more than 7 years. The study population was made up of refugees living in the camp and included in the study were consenting refugees aged between 6 to 80 years and consenting health care workers in the camp. Data was collected using random sampling and the sample size was determined according to the lorentz formula (16).


$${}^{\underset{{\text{E}}^{2}}{\text{n=P}\;\left(100-\text{P}\right)\;{\text{Z}}^{2}}}$$

Where: n = Sample size,

P= % of occurrence of characteristic (50% as estimate is best used),

E= % maximum of required error (5% is the accepted value in social research),

Z= Confidence required (The typical levels of confidence used are 1.96 for 95% confidence and 2.57 for 99%).

Our minimum sample size was 384 refugees.

### Survey instrument

The collection of data was done in 2 phases;

- Data for refugees was collected using a semi-structural open ended and close ended questionnaire and
- The second phase involved the collection of data from the health care workers in the refugee camp

Data for refugee participants were collected in two steps; an anonymous questionnaire was used to collect the personal information of the participants like socio-demographic data (age, sex, profession, education status), past dental history, type of treatment received and impacts of the treatment received and oral health knowledge, practices (Table I).

**Table I:** Evaluation table for refugee dental knowledge

Rank	Mark/10	Percentages
Poor	0-2	0- 20%
Insufficient	3-4	21- 49%
Satisfactory	5-7	50- 70%
Very satisfactory	>7	>70%

Secondly a clinical examination was carried out in a portable dental couch using a dental mirror and probe. Extra oral and intra oral examination were carried out in order to evaluate the oral health status of the refuges and to identify the presence of any functional and structural disorders of the lower and upper jaws, and to detect the occurrence of dental and periodontal pathologies like the dental caries and periodontal disease experiences of the participants and the unmet treatment needs (Table I).

Other oral treatment needs of chronic pathologies such as cellulitis, Trauma etc. including prophylaxis, rehabilitative treatments, were included as Urgent Treatment Needs (Table II). We decided to carry out the impact evaluation based on 3 types of negative effects notably: Physical, social and psychological negative effects. All patients with oral pathologies were referred to the regional hospital for treatment. The second phase of the study involved the collection of data from health care personnel at the refugee camp using a separate questionnaire. Data concerning the oral health care workforce and the technical platform was collected with a data captured sheet using the table below (Table I).

**Table II:** Oral health knowledge, periodontal treatment needs and unmet treatment needs

Oral health knowledge for refugees					
Rank		Mark/10			Percentages
Poor		0-2			0- 20%
Insufficient		3-4			21- 49%
Satisfactory		5-7			50- 70%
Very satisfactory		>7			>70%
**Scoring table of periodontal treatment needs CPITN**					
Indices	Periodontal disease		Code Treatment needs		
Index 0	No periodontal disease		TN 0 No treatment required		
Index 1	Bleeding gum		TN 1 Amelioration of oral hygiene		
Index 2	Presence of tartar		TN 2 Scaling and polishing		
Index 3	Periodontal Pocket of 4-5 mm		TN 3 Intense scaling		
Index 4	Periodontal Pocket >6 mm		TN 4 Intense scaling + surgery		
**Scoring table of dental treatment needs**					
Code	Dental disease			Code	Treatment
0	Healthy tooth			0	No treatment
1	Decayed tooth [categories I II and III]			1	Restorative treatment
2	Total crown destruction			2	Extraction
3	Absent tooth			3	Prosthesis

### Data analysis

Data was captured manually into an excel sheet and was imported to Epi-info 7 for analysis. Test of association was done using Chi Square statistics and P < 0.05 was considered as statistical significance.

### Ethical consideration

Ethical clearance was obtained from the ethical committee of the Faculty of Health Sciences, Université des Montagnes Cameroon (67/CD/UdM/2020). Further, we obtained authorization from the Regional Delegate of public health, East Region and Chief Medical Officer of the health district (Ref/0031/L/Minsante/SC/DRSPE/BFDA).

Each adult participant signed an informed consent form that outlined the rights and responsibilities of the participants while participants below 18 gave their assents with the approval of their parents or guardian to participate in the study. The form included a summary of the purpose of the study and mentioned that their participation is voluntary, with no incentives, and participants could decide to withdraw from the study at any time and reassured participant confidentiality by assuring them of non-disclosure of their personal information.

## Results

A total of 732 participants made up of 716 (97.81%) refugees and 16(2.2%) health personnel were recruited in the study. All the refugees who participated in our study were all of Central African Republic nationality, with an average age of 29.3 ± 14.7 s. d years with ages ranging from 6 to 81 years. Two thirds 314 (61.2%) of the refugees were females, 378 (52.8%) of the refugees were unemployed and 342 (47.8%) married. The majority 701 (97.91%) were Muslims and 511(71.4%) of the participants had no formal education. The majority 707(98.8%) of the participants had lived in the camp between 4 and 6 years and the mean time spent in the refugee camp was 5.9 years ± 0.7s.d years (Table III)

**Table III:** Distribution of refugees according to their socio-demographic profile and professional profile of participant

Age groups (in years)	Frequency	Proportions (%)
6 to 21	220	30.73
20 to 41	339	47.35
40 to 61	127	17.74
60 to 81	30	4.19
Gender		
Male	279	38.8
Female	437	61.2
Occupation		
Jobless/House wife	378	52.8
Employed/Self employed	169	23.6
Scholar	169	23.6
Education level		
No formal education	511	71.37
Primary	154	21.51
Secondary	51	7.12
Marital status		
Married	342	47.77
Divorced	1	0.14
Window (er)	74	10.34
Religion		
Christian	15	2.09
Muslim	701	97.91

### Oral health knowledge

**Figure 1:**
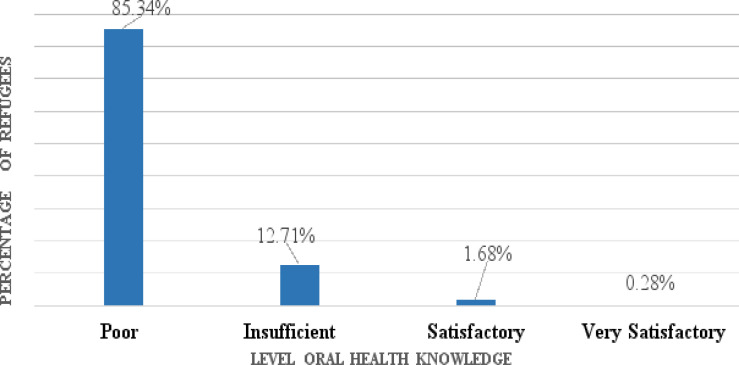
Oral Health Knowledge of refugees

The majority 625 (85.34%) of the participants presented had poor oral health knowledge. The percentage difference was statistically significant with a P-value = 0.002. (Figure 1).

### Oral health seeking behaviour

Though more than a third 286 (40.1%) of refugees had visited a dental facility in the past while 41 (5.7%) had never been to a dental clinic. p-value = 0.001.

Less than half 305 (45.2%) of the refugees who had been to the dental clinic were not satisfied with their treatment (p- value = 0.02). Other methods of satisfactory treatments by the refugees included self-medication 22(3.3%) and African traditional medicine 26(3.9%) (Table IV).

**Table IV:** Management of dental problems

Management of dental problems		Frequency	Percent (%)
No treatment		258	38.22
Health post and satisfied	Yes	17	2.52
	No	305	45.19
Self-medication and satisfied	Yes	22	3.26
	No	11	1.63
African traditional medicine and satisfied	Yes	26	3.85
	No	36	5.33
**Total**		675	100

### Unmet oral health treatment needs

Clinical assessment reveal that the majority 591(82.54%) of the refugees required dental treatments. These include restorative treatment 372(51.9%), tooth extraction 394(55.0), prosthesis 286(39.8%) and periodontal treatments were required by 704 (98.3%) refugees. Only 17.6% of participants needed orthodontic treatments, the percentages difference was statistically significant with a P. value = 0.01. (Table V).

**Table V:** Unmet dental treatment needs and periodontal treatment needs

Dental treatment needs		Frequency	Percent (%)	P value
None	Yes	125	17.46	0.04
	No	591	82.54	
Restorative treatment	Yes	372	51.96	0.91
	No	344	48.04	
Tooth extraction	Yes	394	55.03	0.49
	No	322	44.97	
Orthodontic needs	Yes		17.6	0.01
	No		82.4	
Prosthetic treatment	Yes	286	39.95	0.06
	No	430	60.05	
Periodontal Treatment needs				
None	Yes	12	1.68	0.001
	No	704	98.32	
Oral hygiene amelioration	Yes	452	63.13	0.69
	No	264	36.87	
Scaling	Yes	650	90.78	0.003
	No	66	9.22	
Deep scaling and curettage	Yes	177	24.72	0.04
	No	539	75.28	
Intensive scaling and surgery	Yes	47	6.56	0.02
	No	669	93.44	

Less than a quarter 95(13.3%) refugees needed urgent treatment like dental extractions, the percentages difference was statistically significant with a P. value = 0.001.

### Oral health care facilities and Workforce evaluation

There was no health facility in the health center to cater for the oral health needs of the refugees. The existing health centre was rated poor by 83.1% of the respondents.

We identified 16 health personnel (2.2% of participants) in the health Centre, who gave their consent for our study. Amongst which were general nurses, Midwifes and Nursing aids. There was no facility for dental consultation, restorations and oral surgery.

No oral health campaign had been carried out at the Gado Badzere camp. There were no oral health personnel at the health post.

## Discussions

The current study showed that the oral health status of the refugees was poor, high needs of dental emergencies and the unmet treatment needs was high. The study has shown also that the refugee camp does not have oral health care facilities and personnel to cater for the oral health needs of the refugees.

In the current study, though the population ranged from 6 to 61 years, the mean age current of the study population was 29.3±14.7 years made up of 60% females. This reflects that the wars in the neighbouring countries affected the independent active age group that are supposed to work and cater for their dependents. This younger population mostly made up of women can be trained on basic primary oral health care. Ogunbodede et al. (2000) had suggested that oral health programmes for refugees should emphasize a Primary Health Care approach focusing on prevention, based on appropriate technology, and promoting involvement of the refugee community in the provision of services 17. Training of Refugees as Community Oral Health Workers (COHW) in the Camp will go a long way to reduce the disease burden in the camp. The training and empowerment of refugees on basic oral health care in the camp can be reinforced as half of the refugees were married and most of the refugees jobless. As oral health care workers can easily transmit oral health knowledge to their immediate families and eventually educate the entire population in a shorter time. The current study showed that the oral health knowledge of the population is very low. This is because policy makers did not see the opportunity of the demographic profile of the population as a means of training the population of basic oral health.

Three quarters of the participants of the current study had no formal western education. The reason for this, is that the majority of the refugees being Muslims prefer sending in their kids for koranic education. The poor oral health of the camp cannot be attributed to religion as teachers in Islamic schools can be trained in basic oral health care and this knowledge can be transmitted to the pupils. The fact that majority of the pupils attend Islamic schools si an opportunity for oral health promotion. Almost all the refugees have spent about 6 years in the camp, this can be attributed to the fact the camp experienced a rapid and large influx in 2014 when it was created. This resettlement and cohesion of the refugees for six year means that is the camp is well organised, active oral health education can be carried out by identified leaders of the camp.

As far as oral health care services in the camp is concerned the current study, showed that primary prevention was ineffective with almost all the participants had poor oral health and knowledge. Recent reports have shown that refugees present with poor oral health 13, 14. Poor oral health of refugees is attributable to various factors such as limited access to therapeutic and preventive dental care, lack of dental services in camps, low oral health literacy, cultural and psychological factors 10, limited financial resources, poor diet and limited access to portable water 11, 12. Poor oral hygiene, poor hygiene practices and the interactions of the above factors can aggravate the oral health status of refugees. Geltman et al, 2014 also had similar findings in Massachusetts where 73% of the Somali refugee population had a poor oral health and literacy 18. The majority of the refugees have suffered from at least one oral pathology 18.

Poor oral health amongst refugees may also be a result of a limited diet and lack of access to dental health care in refugee camps and in some cases may also be a result of torture 16

Exposure to western diet rich in sugar at the camp might be responsible for the high caries prevalence. The current study showed that half of the participants in the current study needed tooth extraction and more than a third with prosthetic dental treatment need, an insignificant number of participants requiring intensive scaling and surgical periodontal treatment needs. Though these needs were not urgent, in resource poor settings like the camp we carried out our study, they can be managed using the basic package for oral care. The concept of Basic Package of Oral Care (BPOC) places great emphasis on approaches which are acceptable, feasible and affordable and can be provided within the existing Primary Health Care system in the camp. The most important components of the basic package that could be useful in the camp include Oral Urgent Treatment (OUT), and Atraumatic Restorative Treatment (ART). The successes of these approach depend on prevailing local factors like available human and financial resources, existing infrastructures and community involvement in the primary oral health of community 17.

Though the current study showed that a significant number of participants had urgent treatment needs dental emergencies are very common in refugees' camps. Hamed (2017), Ghiabi (2013) reported dental emergencies in refugees' camps in France and Canada (16,22). Reasons for these emergencies is because of late consultation to the dental services as seen in the current study and might also be that restorative care is not available to the majority of refugees and teeth are left to decay to the extent that they become painful and have to be extracted as the last option. This is common with most economically less developed countries and regions; extraction is the predominant oral care procedure performed by both dentists and dental therapists 17. The current study showed that 11% had urgent treatments needs 17. This could be explained by the fact that NGOs in charge of the health care of the refugees evacuate patients with very critical health pathologies to neighbouring towns with better health facilities for urgent treatment when the dental diseases are in an advanced state. Concerning the impacts of oral health care received in the camp, more than 85% of the refugees presented with at least one negative impact which could be either physical, social, or psychological two or all the three. This with a greater portion presenting severe negative impacts. More specifically concerning oral health, the very important amount of unmet treatment needs showed a great negative impact on the lives of refugees. Our study showed that more than 80% needed dental treatments while about 99% needed periodontal treatment.

According to Geltman (2014), health literacy has a vital role to play in the oral health practices as participants with higher health literacy are 2.0 times more likely to have comply to preventive care (p=0.02) 18. It has been reported that there is a direct relation between the educational level and the oral health knowledge of the refugees 12,18. Hence, we discovered from our study that most of the refugees with none and low level of formal education were amongst those with a poor oral health literacy and presenting with at least an oral pathology.

A study to establish the relationship oral health literacy (OHL) and periodontal diseases revealed that subjects with limited OH level had poorer periodontal compliance 23,^24^. Therefore, improving the OHL of medical instructions, self-management and regions, extraction is the predominant oral care procedure performed by both dentists and dental therapists 17. The current study showed that 11% had urgent treatments needs 17. This could be explained by the fact that NGOs in charge of the health care of the refugees evacuate patients with very critical health pathologies to neighbouring towns with better health facilities for urgent treatment when the dental diseases are in an advanced state. Concerning the impacts of oral health care received in the camp, more than 85% of the refugees presented with at least one negative impact which could be either physical, social, or psychological two or all the three. This with a greater portion presenting severe negative impacts. More specifically concerning oral health, the very important amount of unmet treatment needs showed a great negative impact on the lives of refugees. Our study showed that more than 80% needed dental treatments while about 99% needed periodontal treatment.

According to Geltman (2014), health literacy has a vital role to play in the oral health practices as to preventive care 2.0 times more likely to have comply It has been reported that there is a direct relation between the educational level and the oral health knowledge of the refugees 12,18. Hence, we discovered from our study that most of the refugees with none and low level of formal education were amongst those with a poor oral health literacy and presenting with at least an oral pathology.

A study to establish the relationship diseases revealed that subjects with cigarette smoking the efforts to improve the adherence to and periodontal health and patients may help in skills and the overall treatment outcomes 23. The current study revealed that refugees who had ever suffered from at least one oral pathology were amongst those with poor oral health literacy. The oral health service of the camp was poor due to the absence oral health workers and basic primary health care infrastructure.

The oral health service of the camp was poor due to the absence oral health workers and basic primary health care infrastructure.

A comprehensive primary oral health care programme is essential for refugee camps, especially when the camp has become stable. This unique programme represents an effective approach to oral health promotion in refugee situations characterized by full community participation. Ogunbodede et al., (2000) recommended that UNHCR should accommodate oral health within the major health framework for refugee populations, including provisions for community oral health workers remuneration as well as equipment and material supply 17. The fact that at the camp, there was no oral hygiene workforce and infrastructure, signifies the refugees can be trained to provide preventive oral health care, emergency relief and treatment, and treatment of dental caries using the ART approach 17. Teaching should be focused on the aetiology of oro-facial diseases and oral hygiene instructions. Simple oral hygiene techniques like the use of toothbrushes and chewing sticks should be emphasized during oral hygiene education. Special care should be given to vulnerable groups like expectant mothers, the aged and people with disabilities. Trained refugees also carried out home visits during which information and advice regarding oral health was provided 17.

## Limitations

During the current study, we were confronted to a great language barrier. Almost all the refugees express themselves either in Fulfulde, Sango or Arabic and due to the fact that almost all the refugees had no formal education, it was very tedious to explain each question to each participant during the data collection. Also, many refugees refused to give their consent because of fear of pain during consultations.

Many refugees were ex-combatants in the current war in the Central Africa Republic and had hidden weapons so there was constant risk due to high insecurity in the site.

## Strength

This study gives a picture of many refugee camps in Africa where there is lack of basic health care facilities especially facilities for providing basic oral care. It will contribute to the wealth of knowledge on oral health care of refugees and will encourage further research in this area. It can also be used as a policy document to help improve the oral health accessibility of refugees.

## Conclusions

The prevalence of dental infections was high in the camp with a significant number of refugees presenting with dental emergencies.

The majority of the refugees had a poor oral health literacy level and presented with associated poor oral hygiene. Orofacial pain affected the quality of life of the majority (89%) of the refugees negatively either physically, socially or psychologically

We hence conclude that the oral health care delivered to refugees is of poor quality has influenced the lives of the refugees negatively. The oral health service of the camp was poor due to the absence oral health workers and basic primary health care infrastructure.

## Recommendations

1. The government should establish a primary oral health care facility with personnel with basic infrastructure to compliment the already existing health facility in the camp.

2. Health care personnel working in the refugee camp and some educated refugees should be trained on the basic package of oral health care, which is a cost-effective approach in the management of dental disease.

3. The ministry of health should assign personnel in the already existing oral health care facilities of the region to visit the camp regularly for oral health outreaches so that patients with urgent needs can be screened and referred to the hospitals and also in the reinforcement of oral health education.
